# Swordfish or Shark Slice? A Rapid Response by COIBar–RFLP

**DOI:** 10.3390/foods8110537

**Published:** 2019-11-01

**Authors:** Venera Ferrito, Alessandra Raffa, Luana Rossitto, Concetta Federico, Salvatore Saccone, Anna Maria Pappalardo

**Affiliations:** Department of Biological, Geological and Environmental Sciences, Section of Animal Biology “M. La Greca”, University of Catania, Via Androne 81, 95124 Catania, Italy; vferrito@unict.it (V.F.); alessandra.raffa92@gmail.com (A.R.); lunarossa92@gmail.com (L.R.); federico@unict.it (C.F.); saccosal@unict.it (S.S.)

**Keywords:** COIBar–RFLP (cytochrome oxidase I barcode–restriction fragment length polymorphism), seafood, fraud, DNA barcoding, food authenticity

## Abstract

Market transparency is in strong demand by consumers, and the authentication of species is an important step for seafood traceability. In this study, a simple molecular strategy, COIBar–RFLP (cytochrome oxidase I barcode–restriction fragment length polymorphism), is proposed to unveil commercial fraud based on the practice of species substitution in the swordfish trade. In particular, COI barcoding allowed the identification of the species *Prionace glauca, Mustelus mustelus*, and *Oxynotus centrina* in slices labeled as *Xiphias gladius*. Furthermore, the enzymatic digestion of COI amplicons using the *Mbo*I restriction endonuclease allowed the simultaneous discrimination of the four species. Interestingly, an intraspecific differential *Mbo*I pattern was obtained for the swordfish samples. This pattern was useful to differentiate the two different clades revealed in this species by phylogenetic analyses using several molecular markers. These results indicate the need to strengthen regulations and define molecular tools for combating the occurrence of fraud along the seafood supply chain and show that COIBar–RFLP could become a standardized molecular tool to assess seafood authenticity.

## 1. Introduction

Swordfish fishery is one of the most important fishing activities in the Mediterranean Sea, in particular in South Italy. Quotas have been established to combat overfishing, and fisheries have been closed over several months to protect juveniles. According to recent data from the International Commission for the Conservation of Atlantic Tuna, Italy ranks the highest in terms of swordfish catches, which amount to 45% of the total allowable in the period 2003–2016 [1]. The highest demand for fish products in general, and swordfish in particular, occurs during summer, especially in restaurants [2] but also in local markets. As a result of the high demand, the price of these large pelagic fishes is on average higher than that of small fishes [3]. 

With the increase in demand and price, alimentary fraud potentially increases too. This can include food mislabeling, substitution, counterfeiting, misbranding, dilution, and adulteration [4]. The mislabeling of seafood can be harmful for health, in terms of economic loss, as well as for the loss of biodiversity it may cause in the case of illegal trade of threatened species. For these reasons, European regulations have focused on traceability and, in particular, on the mandatory declaration of the species present in a product on the product label [5,6,7,8,9,10]. 

Despite these adequate legislative tools, the number of cases of food fraud perpetrated in the fish trade in Europe and worldwide is increasing. The results of recent investigations on this phenomenon have shown that the percentage of mislabeling was around 30% of the total samples collected [11,12,13,14,15]. To address this problem, researchers have increasingly asserted the importance of using molecular tools based on DNA sequencing for detecting food fraud. The most common mitochondrial (mt) genes used for this purpose have been cytochrome b, 16S rRNA, and cytochrome oxidase I (COI). Other mtDNA targets, such as the mtDNA control region (CR) [16,17], which has been the most popular molecular marker used for genetic population structure studies [18,19,20,21,22,23,24], have seen limited use in fish and seafood species identification. In recent years, COI has been standardized as a barcode gene for species identification in several animal taxa [25,26,27,28,29,30,31,32,33] including fishes [34,35,36,37,38,39,40]. More specifically, the high number of COI-barcode fish sequences available in the large public gene sequence databases (BOLD and GenBank) [41,42] have made this gene the most highly used gene to clearly identify fish species and cases of mislabeling of seafood products [38,43,44,45,46,47,48,49,50,51,52,53,54]. However, in the context of seafood traceability, the main goal for the implementation of these analyses is to reduce the time it takes from sampling to obtaining gene sequencing results, as well as the costs of processing. 

An already well-proven technique for the identification of species is polymerase chain reaction (PCR)–restriction fragment length polymorphism (RFLP), by which the PCR product of an amplified gene is cut with different restriction endonucleases to obtain a species-specific RFLP [55,56,57], useful for species authentication. In this regard, the combination of DNA barcoding of COI and the consolidated method of RFLP analysis (COIBar–RFLP, cytochrome oxidase I barcode–restriction fragment length polymorphism) has been successfully used to discriminate several fish species belonging to the Engraulidae, Merluccidae, Soleidae, and Acipenseridae families in processed seafood products [49,52,53,54,58]. It should be noted that the time and cost of execution of the COIBar–RFLP are lower than those of DNA sequencing (about 7 h and 10 euros per sample *vs* 24 h and 17 euros per sample, respectively).

Focusing on swordfish adulteration problems, the most commonly used species for fraudulent substitution are elasmobranches, including some species of shark. It should be noted that the market of shark meat is very wide for both fresh and frozen foods also in Italy, and cases of mislabeling have been frequently recorded for these products imported from all over the world [59,60,61]. 

Therefore, food fraud occurs due to an economic return when using shark meat. However, the substitution of a more valuable fish, such as swordfish, with shark meat leads to an even more serious fraud in economic terms. In the last decades, several studies have been carried out to detect the rate of mislabeling of different seafood products, and in some cases, shortfin mako (*Isurus oxyrinchus*) and blue shark (*Prionace glauca*) have been found to be sold as swordfish [36,37,62,63].

On the basis of the considerations above, the aim of this work is to extend the use of COIBar–RFLP to investigate the identity of swordfish products in the south of Italy and to discriminate swordfish (*Xiphias gladius*) from other fish species to detect fraudulent actions, such as species substitution, which represent the most common fraud in seafood. First, we sequenced the conventional COI barcode in a large number of samples collected in local fish markets and supermarkets, labeled as swordfish slices. Subsequently, the COIBar–RFLP procedure was applied on reference samples of the COI-barcoded species to obtain a species-specific restriction enzyme pattern. Finally, this pattern was used for swordfish slice authentication.

## 2. Materials and Methods 

### 2.1. Sampling

Fresh and frozen slices of swordfish were acquired in 2010 and 2018 from local fish markets and supermarkets of south Italy for a total of 35 samples. Another 10 samples from the local harbor were collected and identified on the basis of morphological traits [64,65] and used to construct a reference COI-barcode library. The samples collected in 2010 had already been processed [37] and were used in this study only for the application of COIBar–RFLP. The remaining samples, preserved at room temperature in 1.5 mL labeled tubes filled with 95% ethanol, were processed for DNA barcoding and COIBar–RFLP (Table 1). DNA samples were deposited as vouchers at the Department of Biological, Geological, and Environmental Science, Section of Animal Biology, in Catania, Italy.

### 2.2. DNA Barcoding

Genomic DNA was extracted from 25 mg of tissue using a commercial kit based on silica purification (DNeasy tissue kit, Qiagen, Hilden, Germany) following the manufacturer’s guidelines. All samples were analyzed by amplifying a portion of about 650 bases of the COI gene in a 20 μL reaction mixture also containing the M13 tailed primers (VF2_t1 and FishR2_t1) described in Ivanova et al. [66] to improve the sequencing quality of the PCR products and following the PCR conditions reported by Pappalardo et al. [54]. All PCR products were checked by 0.8% agarose gel electrophoresis, visualized with SYBR^®^ Safe (Thermo Fisher, Waltham, MA USA), displayed through a Safe Imager TM 2.0 Blue Light Transilluminator (Thermo Fisher, Waltham, MA USA), and then purified with the QIAquick PCR purification kit (Qiagen, Hilden, Germany). Sanger sequencing, using M13 primers, was subsequently conducted by Genechron in both forward and reverse directions to generate the DNA barcodes [67].

The sequence chromatograms were checked visually and assembled. Multiple-sequence alignment was carried out by the online version of MAFFT v.7 [68]. Ambiguous sequences were trimmed, and primer sequences were cut. The sequences were carefully checked for the presence of nuclear mitochondrial pseudogenes or NUMTs (nuclear mitochondrial DNA sequences), which could be easily coamplified with orthologous mtDNA sequences [69]. The EMBOSS Transeq tool [70] was used to translate the nucleotide sequences to amino acids to check for premature stop codons and to verify that the open reading frames were maintained in the protein-coding locus. To confirm the identity of the amplified sequences, we conducted BLAST (Basic Local Alignment Search) searches in GenBank with default parameters [71]. All sequences obtained from the present study were published in the National Center for Biotechnology Information database (NCBI), and their GenBank accession numbers are reported in Table 1. After the BLAST search, six shark species sequences (HM909857, JF493927, KF899461, KI709900, JF493694, JN641217) downloaded from GenBank were added to our dataset to construct a phylogenetic tree. We used jModelTest v 2.1.10 [72] to select the best-fitting substitution model for our sequences according to the corrected Akaike information criterion. A maximum likelihood (ML) tree by using a GTR + I + G model was implemented in MEGA v 6.0 (Biodesign Institute, Arizona, MA, USA) [73]. The evaluation of the statistical confidence of nodes was based on 1000 non-parametric bootstrap replicates [74].

### 2.3. COIBar–RFLP

**Figure 1:**
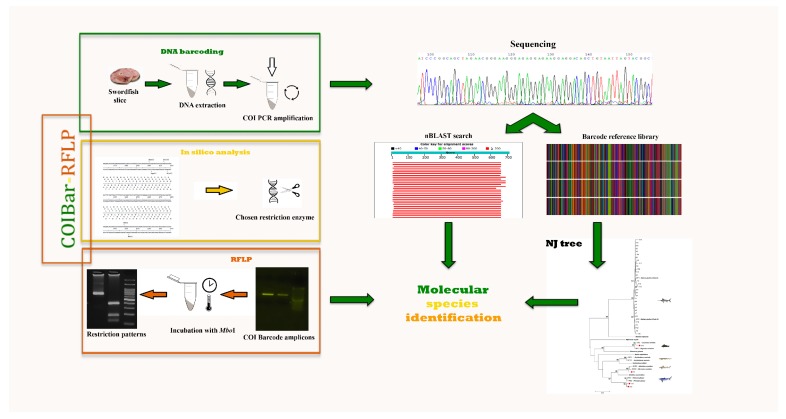
Flow chart of COIBar–RFLP (cytochrome oxidase I barcode–restriction fragment length polymorphism) or species discrimination. DNA barcoding steps: DNA isolation from swordfish slices and barcode region PCR amplification. In silico analysis steps: search for an appropriate restriction enzyme. RFLP steps: incubation of barcode amplicons with *Mbo*I to obtain the COIBar–RFLP pattern. ML, maximum likelihood; nBLAST, nucleotide Basic Local Alignment Search Tool.

The selection of the most suitable restriction enzymes to discriminate swordfish from other shark species (*Mustelus mustelus,* L., 1758, *Oxinotus centrina* (L., 1758), *P. glauca* (L., 1758)*, Scyliorhinus canicula* L., 1758) was performed through “Remap” [75]. The in silico analysis was preliminarily carried out using a total of 10 COI barcode sequences (of about 650 bases) of the examined species, downloaded from public databases (GenBank and BOLD) [41,42]. Five different restriction enzymes were tested to scan all validated sequences and to detect the expected size of the digested products: *Hpa*II (C*CGG), *Hinf*I (G*ANTC), *Mbo*I (*GATC), *Rsa*I (GT*AC), and *Hind*III (A*AGCTT). Finally, a total of 49 COI sequences were analyzed by Remap to test for evidence of intraspecific variation at the recognition site of the restriction endonuclease suitable for simultaneous discrimination of the examined species (Figure 1, Table 2). 

Afterwards, the COI-barcode PCR products obtained from *X. gladius* and shark samples were digested with the selected restriction enzymes. For each endonuclease, a 15 μL reaction volume containing 13 μL of unpurified PCR product, 1 μL of digestion buffer (1X), and 1 μL of each endonuclease (10 U each) was prepared. The reaction mixtures were incubated at an optimum temperature of 37 °C for 1 h. The digested amplicons were then separated on a 3% agarose gel using Trackit TM 100 bp DNA ladder (Invitrogen) as a size standard. The restriction pattern obtained from the validated samples was exploited to unequivocally identify the unknown commercial slices. 

## 3. Results

### 3.1. DNA Barcoding

**Figure 2:**
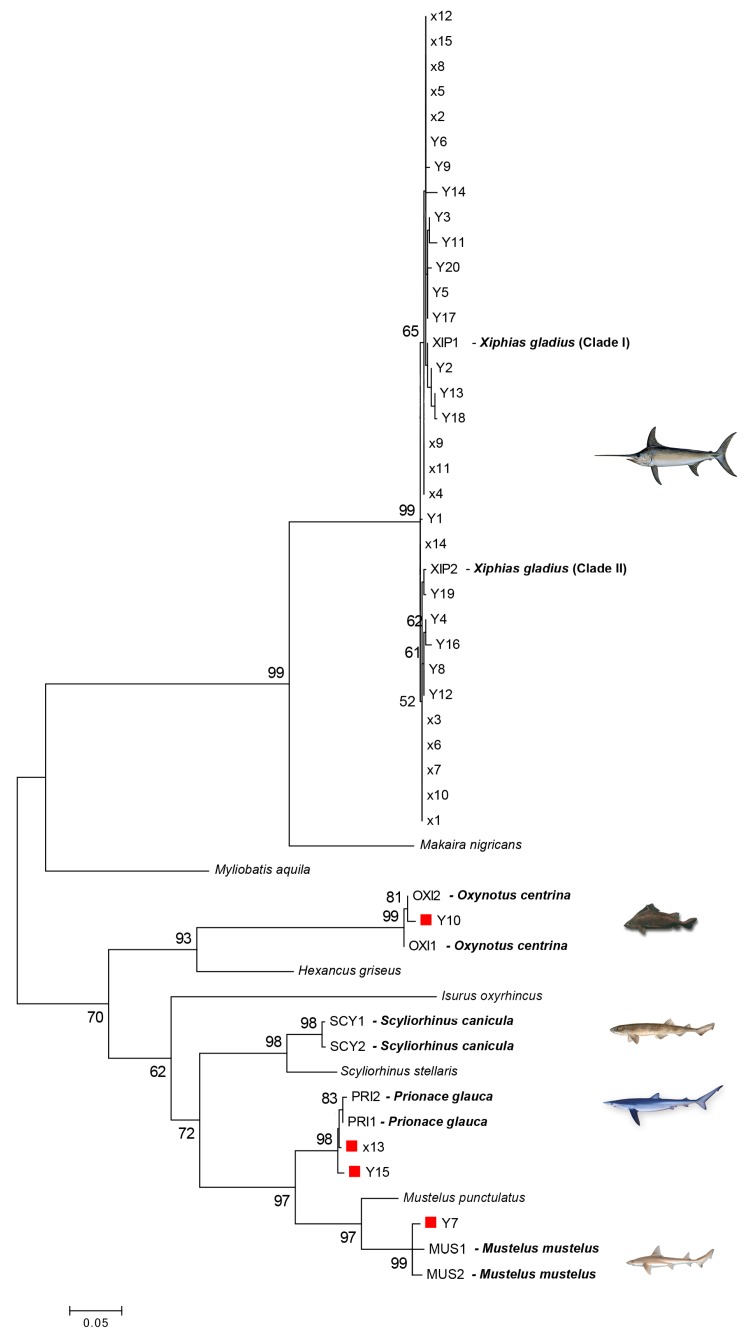
Maximum likelihood (ML) tree showing the relationships of unknown samples sequences (X and Y) to validated reference barcode sequences. The numbers above the nodes represent bootstrap analyses after 1000 replicates. Bootstrap values greater than 60% are shown. The red square indicates swordfish mislabeled samples. Scale bar refers to a distance of 0.05 nucleotide substitutions per site.

The length range of the obtained COI sequences was between 669 bases and 681 bases. Each of them was a functional mitochondrial sequence without stop codons. NUMTs generally smaller than 600 bases were not sequenced [71]. Five species were identified in all examined samples: *X. gladius* (Xiphiidae), *P. glauca* (Charcarinidae), *M. mustelus* (Triakidae), *S. canicula* (Scyliorinidae), and *O. centrina* (Oxynotidae). The sequences obtained from morphologically validated species were compared with the sequences retrieved from GenBank through a BLAST search. The identity percentage between the COI query sequences and their top-match sequences ranged from 98.07% to 100% (Table 1). The ML tree (Figure 2) showed the relationship between the sequences of several unidentified samples and the reference barcode sequences. High bootstrap values (>60%) supported the nodes connecting the sequences of the same species in the tree. The samples of *X. gladius* clustered into two main clades (named clade I and II), as already found by Pappalardo et al. [36,37]. Only one case of mislabeling (1 out of 15) was found in the samples examined in 2010 (6.7%), while 15% (3 out of 20) of mislabeling was found in the samples collected during 2018 (Table 1). Swordfish was substituted with *P. glauca* (2 products), *M. mustelus* (1 product), and *O. centrina* (1 product).

### 3.2. COIBar–RFLP

**Figure 3:**
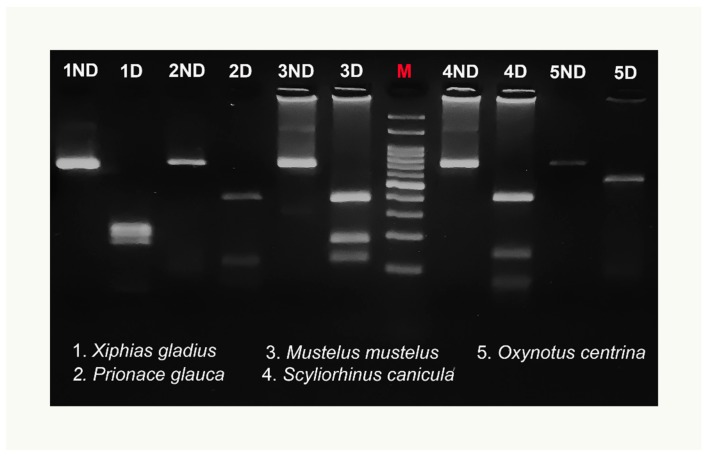
Example of COIBar–RFLP identification of swordfish and shark species on a 3% agarose gel by restriction by *Mbo*I of the cytochrome oxidase I amplicons. Bands smaller than 100 bp were not considered. The 5ND and 5D bands differ in intensity because they were obtained from two different PCR amplifications. ND = not digested, D = digested. M = molecular weight marker (100 bp DNA ladder, biotechrabbit GmbH, Berlin, Germany).

**Figure 4:**
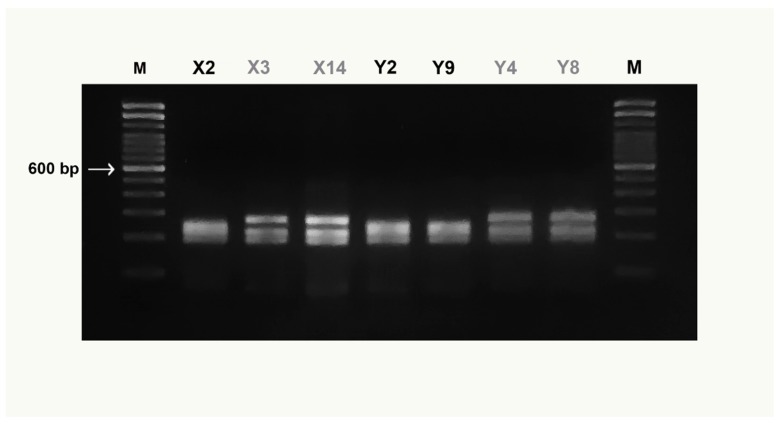
COIBar–RFLP patterns obtained from *Mbo*I digestion of *X. gladius* samples. Clade I (black code), Clade II (grey code). M = molecular weight marker (Trackit ™ 100 bp DNA ladder, Invitrogen). For X and Y codes see Table 1.

The preliminary in silico analysis using “Remap” showed that the *Mbo*I enzyme produced a species-specific pattern useful to discriminate simultaneously all examined species. No intraspecific variation of the *Mbo*I recognition sites was detected for any species tested by “Remap”, with the exception of the *X. gladius* digestion pattern (Table 2). Figure 3 highlights both the size of the undigested COI amplicon, of about 750 bp, and the *Mbo*I differential restriction pattern obtained for each species: one fragment of 510 bp was obtained for *O. centrina*; two fragments of 110 and 400 bp and of 150 and 400 bp were obtained, respectively, for *P. glauca* and *S. canicula*; finally, three fragments of 120, 180, and 390 bp were obtained for *M. mustelus*. The negative control is not shown in the figure. The enzymatic digestion of *X. gladius* amplicons produced two different patterns (Figure 4) corresponding to clades I and II, already described in this species. In particular, three fragments of 170, 220, and 240 bp were detected for clade I and three fragments of 170, 220, and 280 bp were found for clade II. On the basis of this intraspecific pattern, the swordfish sample shown in Figure 3 belongs to clade I.

## 4. Discussion

The results obtained in this study once again confirm the efficacy of COIBar–RFLP in discriminating fish species in commercial products and also highlight the fraudulent practice of species substitutions in seafood products, consisting in the use of less valuable shark species in place of swordfish. The *Mbo*I endonuclease restriction enzyme produced species-specific restriction patterns of the COI amplicons useful to differentiate *X. gladius* from shark species. Another interesting result proving the sensitivity of this methodology is the intraspecific differential *Mbo*I pattern obtained for the swordfish samples. This pattern was useful to discriminate the two different clades revealed in this species by phylogenetic analyses using several molecular markers [36,37,76,77,78]. COI DNA-barcoding showed that 15% of the swordfish samples purchased in local fish markets during 2018 was mislabeled, with an evident economic loss for the consumers. This percentage was at least two times higher than that recorded in 2010, demonstrating that despite the current European legislation focused on consumer protection against fraud, fraud remains frequent and widespread. In this context, there is no doubt that molecular tools are very useful and effective to fight commercial fraud and that DNA-based methods have become increasingly important for seafood authentication. However, while the practice of commercial fraud in the seafood market is a global concern, to date there is no standardized global methodology to expose this practice. Firstly, all states have not yet incorporated into their legislation the use of molecular methods to combat commercial fraud; this is true for Italy, for example. Secondly, significant differences among countries have been found in methods used by accredited laboratories for food authenticity [79]. Thirdly, together with the classic methods (protein- and DNA sequence-based methods), new and sophisticated methods are being developed to identify seafood species [80]. It is evident that the first two issues can be solved only by adopting a common global policy to fight food fraud. The European legislation, for example, could require, rather than only suggest, the application of DNA analysis in the context of seafood traceability [81], also indicating the most useful methodology to be used across European laboratories. In this regard, the features that molecular methods should have for a rapid authentication of species in seafood products can be debated. To be effective for routine activities carried out by local food safety and quality authorities, from the traceability of the catch to the labeling of the products, effectiveness in terms of cost and time-saving and correctness of species identification should be a priority. Among the classic methods, the protein-based methods, such as isoelectric focusing of sarcoplasmic proteins, are still used as official methods for fish species identification [82], but the DNA-sequencing methods, and the DNA-barcoding methodology in particular, have become more common in laboratories specialized in food authentication ([3] and literature therein). Increasingly, new methodologies are emerging for species identification, such as qPCR, DNA microarrays, high-resolution melting analysis, mass spectrometry, high-throughput sequencing, and the recently developed handheld testing devices [80], all of them suitable and effective in terms of cost and time consumption. 

However, these new methodologies require, in some cases, extensive technical equipment and specific skills by the operators and need to be standardized for use as official methods. Furthermore, the application of these methods is limited to a few cases of species authentication, while wide databases of reference samples are needed for their validation as official methods. The methodological approach we propose, COIBar–RFLP, although it cannot substitute DNA sequencing in general, takes advantage of large databases of reference DNA sequences of fish species and of the positive results from several study cases for species of relevant commercial interest under various food matrices [49,52,53,54,58]. COIBar–RFLP successfully and simultaneously discriminated the fish species analyzed in these studies, through the banding pattern obtained after digestion with only one endonuclease restriction enzyme. This simple, robust, easy-to-perform, and cost-effective strategy can potentially cover a wide range of species and provide a versatile tool to monitor the mislabeling of fish products. However, it should be noted that poor enzyme storage, as well as the processing conditions, could compromise the advantages of the methodology in terms of expected time of processing and misleading results. In a recent investigation on the methodological approach performed in 45 European laboratories, Griffiths et al. [79] revealed that PCR–RFLP was used in 40% of the laboratories involved in seafood authentication; this result suggests that this method could become a standardized molecular tool to assess seafood authenticity. 

## 5. Conclusions

The efficacy of COIBar–RFLP was tested for species authentication on slices labeled as swordfish. The illegal practice of species substitution was observed, with the species *P. glauca*, *M. mustelus*, and *O. centrina* being sold in place of swordfish. These results indicate the need to strengthen regulations and to define molecular tools to fight the occurrence of fraud along the seafood supply chain, from the traceability of the catch to the labeling of the products, and to achieve market transparency, which is highly demanded by the consumers. Finally, the future perspectives of COIBar–RFLP rest on the need to build a database of COI restriction patterns to be used for unequivocal species identifications.

## Figures and Tables

**Table 1:** Samples examined in this study.

Code	DNA Sample Voucher	Declared Species	GenBank Access N°	Species Matched by BLAST	Matched Accession From BLAST	% Identity With 100% Coverage
XIP1	DBGES18-125	Xiphias gladius *	JN083390	*Xiphias gladius*	JN083390	100
XIP2	DBGES18-129	Xiphias gladius *	MN447670	*Xiphias gladius*	JN049558	99.71
MUS1	DBGES18-133	Mustelus mustelus *	MN447688	*Mustelus mustelus*	JN641215	99.39
MUS2	DBGES18-134	Mustelus mustelus *	MN447689	*Mustelus mustelus*	JN641214	99.24
OXY1	DBGES18-111	Oxynotus centrina *	MN447691	*Oxynotus centrina*	JF834320	98.96
OXY2	DBGES18-130	Oxynotus centrina *	MN447692	*Oxynotus centrina*	JF834320	98.66
PRI1	DBGES18-121	Prionace glauca *	MN447694	*Prionace glauca*	KJ146044	99.85
PRI2	DBGES18-138	Prionace glauca *	MN447695	*Prionace glauca*	MH719984	99.83
SCY1	DBGES18-144	Scyliorhinus canicula *	MN457949	*Scyliorhinus canicula*	KJ205311	99.70
SCY2	DBGES18-146	Scyliorhinus canicula *	MN457950	*Scyliorhinus canicula*	KJ205311	99.85
X1	DBGES10-045	*Xiphias gladius*	JN083389	*Xiphias gladius*	JN083389	100
X2	DBGES10-048	*Xiphias gladius*	JN083387	*Xiphias gladius*	JN083387	100
X3	DBGES10-049	*Xiphias gladius*	JN049559	*Xiphias gladius*	JN049559	100
X4	DBGES10-054	*Xiphias gladius*	JN083397	*Xiphias gladius*	JN083397	100
X5	DBGES10-055	*Xiphias gladius*	JN083387	*Xiphias gladius*	JN083387	100
X6	DBGES10-058	*Xiphias gladius*	JN049559	*Xiphias gladius*	JN049559	100
X7	DBGES10-059	*Xiphias gladius*	JN049559	*Xiphias gladius*	JN049559	100
X8	DBGES10-060	*Xiphias gladius*	JN083387	*Xiphias gladius*	JN083387	100
X9	DBGES10-068	*Xiphias gladius*	JN083393	*Xiphias gladius*	JN083393	100
X10	DBGES10-074	*Xiphias gladius*	JN049559	*Xiphias gladius*	JN049559	100
X11	DBGES10-077	*Xiphias gladius*	JN083393	*Xiphias gladius*	JN083393	100
X12	DBGES10-080	*Xiphias gladius*	JN083387	*Xiphias gladius*	JN083387	100
X13	DBGES10-083	*Xiphias gladius*	MN447696	*Prionace glauca*	MH719984	99.83
X14	DBGES10-089	*Xiphias gladius*	JN083386	*Xiphias gladius*	JN083386	100
X15	DBGES10-095	*Xiphias gladius*	JN083387	*Xiphias gladius*	JN083387	100
Y1	DBGES18-132	*Xiphias gladius*	MN447671	*Xiphias gladius*	JN049558	99.71
Y2	DBGES18-135	*Xiphias gladius*	MN447672	*Xiphias gladius*	JN083390	99.71
Y3	DBGES18-136	*Xiphias gladius*	MN447673	*Xiphias gladius*	JN083387	99.71
Y4	DBGES18-137	*Xiphias gladius*	MN447674	*Xiphias gladius*	JN049558	99.71
Y5	DBGES18-139	*Xiphias gladius*	MN447675	*Xiphias gladius*	JN083387	99.85
Y6	DBGES18-141	*Xiphias gladius*	MN447676	*Xiphias gladius*	JN083387	99.71
Y7	DBGES18-143	*Xiphias gladius*	MN447690	*Mustelus mustelus*	JN641214	99.39
Y8	DBGES18-144	*Xiphias gladius*	MN447677	*Xiphias gladius*	JN049558	99.85
Y9	DBGES18-151	*Xiphias gladius*	MN447678	*Xiphias gladius*	JN083387	99.71
Y10	DBGES18-152	*Xiphias gladius*	MN447693	*Oxynotus centrina*	JF834320	98.07
Y11	DBGES18-153	*Xiphias gladius*	MN447679	*Xiphias gladius*	JN083387	99.12
Y12	DBGES18-154	*Xiphias gladius*	MN447680	*Xiphias gladius*	JN049558	99.85
Y13	DBGES18-161	*Xiphias gladius*	MN447681	*Xiphias gladius*	JN083390	99.41
Y14	DBGES18-162	*Xiphias gladius*	MN447682	*Xiphias gladius*	JN083387	98.97
Y15	DBGES18-172	*Xiphias gladius*	MN447697	*Prionace glauca*	MH194484	99.39
Y16	DBGES18-173	*Xiphias gladius*	MN447683	*Xiphias gladius*	JN049558	99.27
Y17	DBGES18-174	*Xiphias gladius*	MN447684	*Xiphias gladius*	JN083387	99.85
Y18	DBGES18-175	*Xiphias gladius*	MN447685	*Xiphias gladius*	JN083390	99.27
Y19	DBGES18-182	*Xiphias gladius*	MN447686	*Xiphias gladius*	JN049558	99.71
Y20	DBGES18-187	*Xiphias gladius*	MN447687	*Xiphias gladius*	JN083387	99.56

DBGES (Department of Biological, Geological and Environmental Sciences). BLAST (Basic Local Alignment Search Tool). * = species of the samples identified by using morphological features.

**Table 2:** In silico analysis of swordfish and shark COI sequences scanned by Remap and using MboI as restriction endonuclease.

Species	Sequence Number	Genbank Accession Number	Sequence Size (bases)	Restriction Fragment Size (base pair)
*Xiphias gladius*	10	JN083387	682	≈ 145 - 270 - 220
		JN083389	682	≈ 145 - 270 - 220
		JN049558	682	≈ 145 - 270 - 220
		JF952886	652	≈ 145 - 265 - 220
		HQ024928	652	≈ 145 - 265 - 220
		HQ024927	652	≈ 145 - 265 - 220
		KR086931	652	≈ 145 - 265 - 220
		GU324195	652	≈ 145 - 265 - 220
		DQ107625	655	≈ 145 - 265 - 200
		DQ107623	655	≈ 145 - 265 - 220
*Mustelus mustelus*	10	JN641215	676	≈ 80 - 390 - 171
		JN641214	679	≈ 80 - 390 - 169
		JN641213	672	≈ 80 - 390 - 167
		JN641212	666	≈ 80 - 390 - 156
		JN641211	681	≈ 80 - 390 - 170
		KJ768265	652	≈ 80 - 390 - 142
		KJ768266	652	≈ 80 - 390 - 142
		JN641208	656	≈ 75 - 390 - 156
		JN641209	669	≈ 80 - 390 - 160
		JN641210	664	≈ 80 - 390 - 160
*Oxynotus centrina*	9	KT307360	648	≈ 510 - 95
		KT307361	620	≈ 480 - 95
		KT307362	648	≈ 510 - 95
		KT307363	648	≈ 510 - 95
		KT307364	648	≈ 510 - 95
		JF834320	672	≈ 505 - 100
		KY176547	642	≈ 495 - 105
		GU805137	637	≈ 500 - 95
		GU805138	648	≈ 510 - 95
*Prionace glauca*	10	JN312505	652	≈ 85 - 405 - 70
		JN312504	652	≈ 85 - 405 - 70
		JN312503	652	≈ 85 - 405 - 70
		KP193446	652	≈ 85 - 405 - 70
		KP193455	652	≈ 85 - 405 - 70
		KP193350	652	≈ 85 - 405 - 70
		KP193339	652	≈ 85 - 405 - 70
		KP193159	652	≈ 85 - 405 - 70
		KC015834	652	≈ 85 - 405 - 70
		KC015833	652	≈ 85 - 405 - 70
*Scyliorhinus canicula*	10	JN641243	675	≈ 85 - 400 - 100
		JN641242	676	≈ 85 - 400 - 100
		JN641241	652	≈ 70 - 400 - 100
		JN641240	680	≈ 85 - 400 - 100
		JN641239	675	≈ 85 - 400 - 100
		JN641238	671	≈ 85 - 400 - 100
		JN641237	680	≈ 85 - 400 - 100
		JN641236	674	≈ 85 - 400 - 100
		JN641234	672	≈ 85 - 400 - 100
		JN641233	680	≈ 85 - 400 - 100
